# Primary microRNA processing is functionally coupled to RNAP II transcription *in vitro*

**DOI:** 10.1038/srep11992

**Published:** 2015-07-07

**Authors:** Shanye Yin, Yong Yu, Robin Reed

**Affiliations:** 1 Department of Cell Biology, Harvard Medical School, 240 Longwood Ave. Boston MA 02115.

## Abstract

Previous studies ***in vivo*** reported that processing of primary microRNA (pri-miRNA) is coupled to transcription by RNA polymerase II (RNAP II) and can occur co-transcriptionally. Here we have established a robust ***in vivo*** system in which pri-miRNA is transcribed by RNAP II and processed to pre-miRNA in HeLa cell nuclear extracts. We show that both the kinetics and efficiency of pri-miRNA processing are dramatically enhanced in this system compared to that of the corresponding naked pri-miRNA. Moreover, this enhancement is general as it occurs with multiple pri-miRNAs. We also show that nascent pri-miRNA is efficiently processed before it is released from the DNA template. Together, our work directly demonstrates that transcription and pri-miRNA processing are functionally coupled and establishes the first ***in vivo*** model systems for this functional coupling and for co-transcriptional processing.

It is now well established that transcription by RNA polymerase II (RNAP II) is coupled to the different steps in RNA processing, including capping, splicing, and polyadenylation, via an extensive network of both physical and functional interactions^1^,^2^,^3^,^4^,^5^,^6^,^7^,^8^,^9^,^10^,^11^. Coupling typically results in a potent increase in the rate and efficiency of the RNA processing reactions and is thought to enhance the overall fidelity and efficiency of gene expression. In addition, the majority of nascent transcripts undergo RNA processing co-transcriptionally^12^,^13^, meaning that processing occurs while the transcripts are still tethered to RNAP II. In our previous work, we established a robust *in vitro* system in which transcription is coupled to splicing^14^, and a 3-way system for coupling transcription to both splicing and polyadenylation^15^,^16^,^17^. In addition, we previously established a model *in vitro* system for co-transcriptional splicing in which splicing occurs while the nascent pre-mRNA transcript is tethered to RNAP II^18^.

In the present study, we focused on miRNA processing. MiRNAs are small, non-coding RNAs that play critical roles in post-transcriptional control of gene expression^19^,^20^. In mammalian cells, most miRNAs are transcribed by RNAP II as primary miRNAs (pri-miRNAs)^19^, which range from a few hundred nucleotides (nts) to several kilobases. Some miRNAs are transcribed by their own promoter but the majority are found within introns^20^. Pri-miRNAs are first processed in the nucleus by the microprocessor (Drosha and DGCR8) into ~70 nt hairpin precursor miRNAs (pre-miRNAs) and the 5′ and 3′ flanking regions^21^,^22^,^23^. After processing, pre-miRNAs are exported to the cytoplasm^24^ where they undergo further processing by Dicer to yield ~22 nt mature miRNAs^25^. Mature miRNAs are then assembled into RNA induced silencing complexes (RISC) and guided to the 3′ UTR of target mRNAs to suppress gene expression by translation inhibition and/or mRNA degradation^25^,^26^.

Most pri-miRNAs are transcribed by RNAP II and are also capped and polyadenylated^27^, raising the possibility that processing of pri-miRNA transcripts, like their pre-mRNA counterparts, can occur co-transcriptionally and is also coupled to RNAP II transcription^28^. Indeed, *in vivo* evidence has been obtained for both events. Co-transcriptional processing is supported the observation that an intronic pri-miRNA is processed prior to splicing^29^ and by the observation that cleavage of pri-mRNA by the microprocessor results in attenuated transcription downstream of the pre-miRNA^30^. In addition, chromatin immunoprecipitation studies revealed that the microprocessor is associated with pri-miRNA genomic loci, revealing that the microprocessor is present at sites of active pri-miRNA transcription^29^. Moreover, endogenous pri-miRNAs were present in chromatin-associated nuclear fractions, consistent with co-transcriptional processing^29^,^31^,^32^. *In vivo* studies have also provided evidence that pri-miRNA processing is coupled to transcription. Specifically, when a pri-miRNA lacking a 3′-end cleavage and polyadenylation (CPA) signal was expressed in HeLa cells, the pri-mRNA was retained on the DNA template and processed with higher efficiency than pri-miRNA that was capable of undergoing CPA and release from the DNA^31^,^32^.

Although *in vivo* studies indicate that pri-miRNA processing is both coupled to transcription and occurs co-transcriptionally, the steps of the pri-miRNA processing reaction itself were elucidated using *in vitro* systems. In these systems naked pri-miRNA transcripts generated by T7 polymerase are processed in cell lysates or by purified Drosha/DGCR8^21^,^22^,^23^. To understand the mechanisms underlying coupling and co-transcriptional processing, *in vitro* systems that recapitulate these events are required. Here, we have established a robust *in vitro* system in which pri-miRNA is generated by RNAP II transcription and processed in HeLa cell nuclear extracts. This system faithfully recapitulates the steps in pri-miRNA processing, and we show that both the kinetics and efficiency of pri-miRNA processing are dramatically enhanced relative to processing of the corresponding naked T7 transcripts. Moreover, using an immobilized template assay, we show that Drosha cleavage occurs prior to release of the nascent pri-miRNA from the DNA template. Together, our results directly demonstrate coupling between transcription and pri-miRNA processing and establish the first *in vitro* systems for this coupling and for processing while the nascent pri-miRNA is tethered to the DNA template.

## Results and Discussion

### Establishing a system for RNAP II transcription and processing of pri-miRNA ***in vitro***

**Figure 1:**
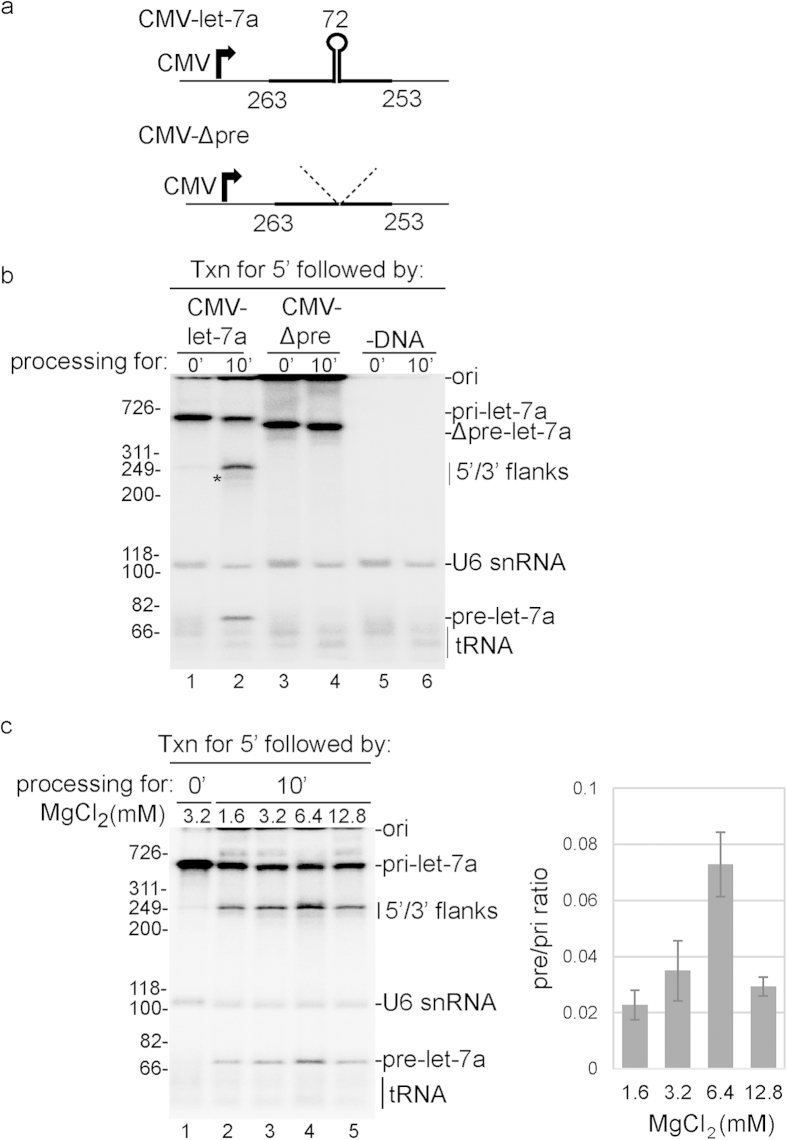
RNAP II txn/pri-miRNA processing system *in vitro*. (**a**) Structure of the CMV DNA template encoding let-7a pri-miRNA used for txn/pri-miRNA processing. The sizes of 5′ flanking region, 3’ flanking region, and pre-let-7a are indicated. The thick line indicates the natural pri-miRNA sequences and the thin lines indicate the vector sequences. CMV-Δpre-let-7a is a mutant lacking the 72 nt pre-let-7a hairpin. (**b**) RNAP II txn/pri-miRNA processing was carried out for the indicated times using the CMV DNA template encoding let-7a pri-miRNA (lanes 1, 2), the CMV DNA template encoding let-7a Δpre-miRNA DNA template (lanes 3, 4) or no DNA template (lanes 5, 6). The asterisk indicates the 3′ flanking region, which lacks a cap and is therefore largely degraded during the reaction (lane 2). (**c**) RNAP II txn/pri-miRNA processing was carried using different amounts of MgCl_2_ as indicated and the CMV DNA template encoding let-7a pri-miRNA. Size markers (in base pairs) and RNA species are indicated. Ori indicates the gel origin. RNAs were detected by phosphorimager and quantified using Quantity One software. Mean ± S.D. of three biological replicates are shown.

**Figure 2:**
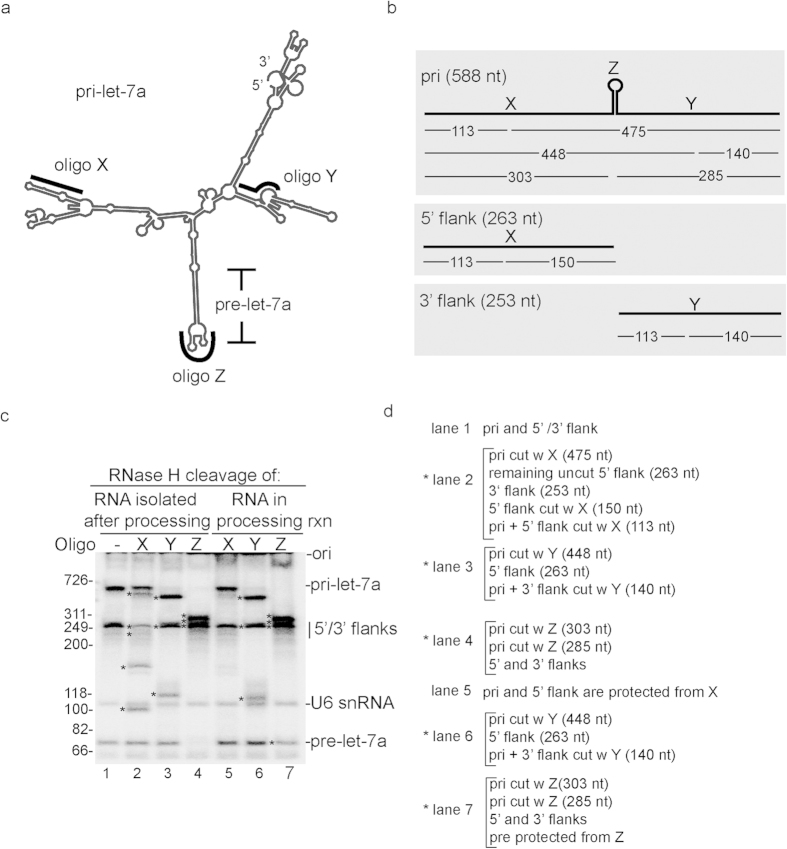
Identification of RNA species generated in the RNAP II txn/processing system. (**a**) Predicted secondary structure of let-7a pri-miRNA showing the binding sites of the X, Y and Z DNA oligos used for oligonucleotide-directed RNAse H cleavage. (**b**) Schematic of let-7a pri-miRNA showing RNAse H cleavage sites in the 5′ flanking region, 3′ flanking region, and pre-let-7a loop using the X, Y and Z DNA oligos. (**c**) No oligo (lane 1) or the indicated oligos (lanes 2–7) were used for RNAse H cleavage of total RNA isolated from the txn/pri-miRNA processing system (lanes 2–4) or carried out directly in the txn/pri-miRNA processing system after completion of the reaction (at the 10 min time point). The RNAse H cleavage products or pri-miRNA processing products are indicated by an asterisk to the left of the gel lanes and are described in panel **d** from top to bottom for each gel lane in panel **c**.

In previous *in vitro* studies, pri-miRNA processing was carried out in total cell lysates or nuclear extracts using naked pri-miRNA transcripts^21^,^22^,^23^. Although pri-miRNAs were processed, the efficiency was low. Consistent with these studies, we found a low level of processing of naked T7 pri-let-7a in HeLa cell nuclear extract (Supplementary Fig. S1a,b). In previous work, we found that pre-mRNA splicing was potently enhanced when nascent pre-mRNA was transcribed by RNAP II^14^. As miRNAs are also synthesized by RNAP II, we asked whether transcription by RNAP II affects pri-miRNA processing *in vitro*. To do this, we constructed a DNA template driven by the CMV promoter and encoding let-7a pri-miRNA (Supplementary Fig. S2a). For comparison, we used a naked T7 transcript encoding let-7a pri-miRNA. We first tested our RNAP II transcription/splicing (txn/splicing) conditions for pri-miRNA processing. This reaction mixture contains nuclear extract, ^32^P-UTP, 3.2 mM MgCl_2_, ATP, and an ATP regenerating system (see Methods)^14^. We carried out these reactions in both the presence and absence of the crowding agent PVA, which is known to enhance gene expression steps *in vitro*^15^,^33^ (Supplementary Fig. S2b,c). In addition, we tested whether formation of an RNAP II pre-initiation complex (PIC)^18^,^33^ stimulated pri-mRNA processing (Supplementary Fig. S2d,e). Both U6 snRNA and tRNA are known to be labeled by ^32^P-UTP in our coupled txn/splicing system^14^,^34^, and both of these RNA species were detected under all conditions we tested (Supplementary Fig. S2b–d). When we used PIC conditions in the presence of PVA, we observed a large increase of RNAP II transcription, and faint bands that could correspond to pri-miRNA processing products were also detected (Supplementary Fig. S2d). Thus, we further optimized this system. To do this, we carried out transcription for 5 min using PIC conditions with PVA, diluted this reaction four fold into fresh nuclear extract, followed by continued incubation for 0 or 10 min at 37 °C (Fig. 1b, lanes 1 and 2). Strikingly, the combination of PIC formation in the presence of PVA and addition of fresh nuclear extract led to a potent enhancement of let-7a pri-miRNA processing. At the 10-min time point, bands of the expected sizes for the 5′ and 3′ flanks (263, 253 nts, respectively, Fig. 1a) were detected (Fig. 1b, lane 2). The putative 3′ flank (designated by *) is largely degraded, most likely because it lacks a 5′ cap. A band of the expected size for the pre-let-7a miRNA was also observed (72 nts, Fig. 2b, lane 2; and see below for identification of processing products). The putative processing is specific as it was not observed when we used CMV-Δpre-let-7a, a construct in which the 72 nt stem-loop of the pre-miRNA was deleted (see schematic in Fig. 1a and Fig. 1b, lanes 3 and 4) or when DNA was omitted from the reaction mixture (Fig. 1b, lanes 5 and 6). In contrast to the efficient processing observed with this system, there was no significant effect on processing of naked T7 pri-miRNA let-7a under the same conditions (see below). We also determined the optimal MgCl_2_ concentration for the txn/pri-miRNA processing system. Consistent with the naked T7 pri-miRNA processing system, we found that the optimal concentration is 6.4 mM, but processing was still efficient at all concentrations tested within the range of 1.6 mM to 12.8 mM (Fig. 1c, lanes 2–5).

### Identification of let-7a pri-miRNA processing products

To identify the RNA species generated in the txn/pri-miRNA processing system, we performed oligonucleotide-directed RNase H cleavage assays. Three oligos, X, Y, and Z, which target the 5′ flank, the 3′ flank, and the loop, respectively, were used for this analysis. The predicted secondary structure of pri-let-7a by CONTRAfold^35^ and the binding sites of the oligos are shown in Fig. 2a, and the predicted sizes of cleaved products are shown in Fig. 2b. We first performed RNase H cleavage of total RNA isolated from the txn/pri-miRNA processing reaction (Fig. 2c, lanes 1–4). The RNA species in each of the gel lanes are explained from top to bottom in Fig. 2d, and the asterisks to the left of each gel lane (Fig. 2c) indicate the cleavage products explained in Fig. 2d. When oligonucleotide X was used for cleavage, the pri-miRNA and 5′ flank were both (partially) cleaved to generate the expected ~113 nt band (Fig. 2b,c, lane 2). We also detected the ~475 and ~150 nt bands from the pri-miRNA and 5′ flank, respectively (Fig. 2b,c, lane 2). Cleavage of the pri-miRNA and 5′ flank with oligo X may have been only partial because of the strong secondary structure formed at the oligo binding site (Fig. 2a). The 3′ flank was not cut with oligo X as expected (Fig. 2b,c, lane 2). When oligo Y was used, the pri-miRNA was completely cleaved to generate ~448 and ~140 nt bands (Fig. 2b,c, lane 3). In addition, the predicted 3′ flank was no longer detected, indicating that it was cleaved. The expected ~113 nt band from the 3′ flank was not detected most likely due to the minor amounts of 3′ flank that are present due its degradation during the pri-miRNA processing reaction. As predicted, the 5′ flank was not cleaved by oligo Y (Fig. 2a,c, lane 3). Finally, when the Z oligo was used, the pri-miRNA was cleaved to generate expected bands of ~285 and ~303 nts (Fig. 2b,c, lane 4). In addition, the 72 nt pre-let-7a was cleaved, and no bands were detected after cleavage of this small RNA (Fig. 2b,c, lane 4). Together, these data indicate that the expected pri-miRNA processing products are faithfully generated in the txn/pri-miRNA processing system.

We next asked whether there were any changes in the RNAse H cleavage products when the oligos were added directly to the reaction mixture after processing for 10 min (Fig. 2c, lanes 5–7). This analysis revealed that oligo X did not cut at all (Fig. 2c, compare lanes 1 and 5), indicating that the structure and/or factors bound to the RNA are masking this region of the pri-miRNA and the 5′ flank. In contrast, cleavage using oligo Y generated RNA species that were similar to those observed with the naked RNA isolated from the reaction (Fig. 2c, compare lanes 3 and 6), indicating that this region is accessible to cleavage in the txn/pri-miRNA processing reaction. When oligo Z was added to the txn/pri-miRNA processing system, the pri-miRNA was cleaved to completion as observed with the naked RNA (Fig. 2b,c, compare lanes 4 and 7). In marked contrast, the terminal loop of pre-let-7a in the same reaction was protected from RNAse H cleavage, which was not the case with the naked pre-miRNA isolated after the txn/pri-miRNA processing reaction (Fig. 2b,c, compare lanes 4 and 7). This result indicates that the terminal loop is bound to a factor and/or has an altered structure after pri-miRNA processing. This alteration to the loop may indicate that the pre-miRNA is bound by machinery involved in, for example, pre-miRNA export or subsequent processing of the pre-miRNA by dicer.

### RNAP II transcription is functionally coupled to pri-miRNA processing

**Figure 3:**
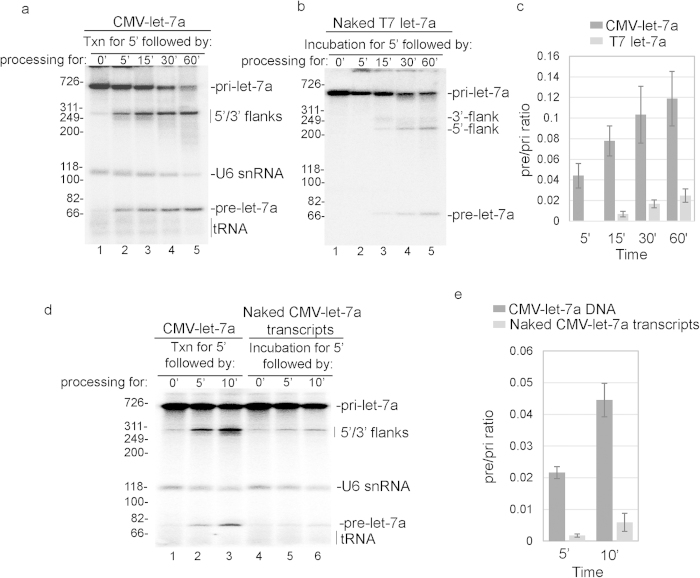
Let-7a pri-miRNA processing is functionally coupled to RNAP II transcription. (**a,b**) CMV DNA template encoding let-7a pri-miRNA (**a**) or the corresponding T7 let-7a pri-miRNA (**b**) was incubated in nuclear extract in the txn/pri-miRNA processing system for the indicated times. Markers (in base pairs) and RNA species are indicated. (**c**) RNAs were detected by phosphorimager and quantified using Quantity one software. The ratios of pre-let-7a signal at each point (lanes 2–5) to pri-let-7a signal at the start of the reaction (lane 1) were calculated and mean ± S.D. of three biological replicates are shown. The difference in pre/pri ratio between the two groups at each time point was analyzed by Student’s t-test.(**d**) CMV DNA template encoding let-7a pri-miRNA (lanes 1–4) or naked CMV let-7a pri-miRNA (lanes 5–8) was incubated in nuclear extract under txn/pri-miRNA reaction conditions for the indicated times. Markers (in base pairs) and RNA species are indicated. Ori marks the gel origin. (**e**) Same as **c** except that the data in panel **d** were quantitated. Mean ± S.D. of three biological replicates are shown, and the difference in pre/pri ratio between the two groups was examined using Student’s t-test.

We next investigated the efficiency and kinetics of pri-miRNA processing in our txn/pri-miRNA system versus processing of naked pri-miRNAs. To do this, we first carried out a time course comparing txn/pri-miRNA processing using CMV pri-let-7a DNA versus naked T7 pri-let-7a (Fig. 3a–c). This analysis revealed that processing of the RNAP II-generated transcript occurred as early as 5 min of incubation and continued to increase (Fig. 3a). In marked contrast, processing of naked T7 pri-let-7a incubated under the same conditions did not even begin until the 15 min time point (Fig. 3b) and the yields of pre-let-7a were significantly lower (Fig. 3c, p < 0.001, student’s t-test). We conclude that processing of pri-let-7A miRNA in the txn/pri-miRNA system occurs with faster kinetics and efficiency than that of naked T7 pri-miRNA transcripts under the same conditions.

To investigate the possibility that the RNAP II-generated transcript is different in some regard from the corresponding T7 transcript, such as RNA modifications that could facilitate processing, we generated RNAP II let-7a pri-miRNA using our PIC/txn conditions and isolated total RNA from the reaction mixture. We then compared processing of this naked pri-let-7a transcript to that of its counterpart generated by RNAP II transcription in the txn/pri-miRNA processing system (see Methods). This analysis revealed that rapid and efficient processing only occurred when the CMV let-7a pri-miRNA was synthesized during the txn/pri-miRNA processing reaction and not with the identical naked CMV let-7a pri-miRNA (Fig. 3d,e, p < 0.001, student’s test). Together, these data showing that transcription by RNAP II potently enhances both the kinetics and efficiency of pri-miRNA processing, indicating that RNAP II transcription is functionally coupled to processing, and our system recapitulates this coupling *in vitro*.

### Functional coupling of transcription to pri-miRNA processing is general

To investigate the generality of our results obtained with let-7a pri-miRNA, we examined two additional pri-miRNAs (Supplementary Fig. S3). This analysis showed rapid and efficient processing in the txn/pri-miRNA processing system with two different CMV pri-miRNA constructs, one encoding miR-21 and the other encoding miR-26. In contrast, their corresponding T7 transcript counterparts were not efficiently processed (Supplementary Fig. S3). We conclude that functional coupling of transcription to pri-miRNA processing is general and we will refer to this as the coupled txn/pri-miRNA processing system.

### Efficient txn/pri-miRNA processing occurs on an immobilized DNA template

**Figure 4:**
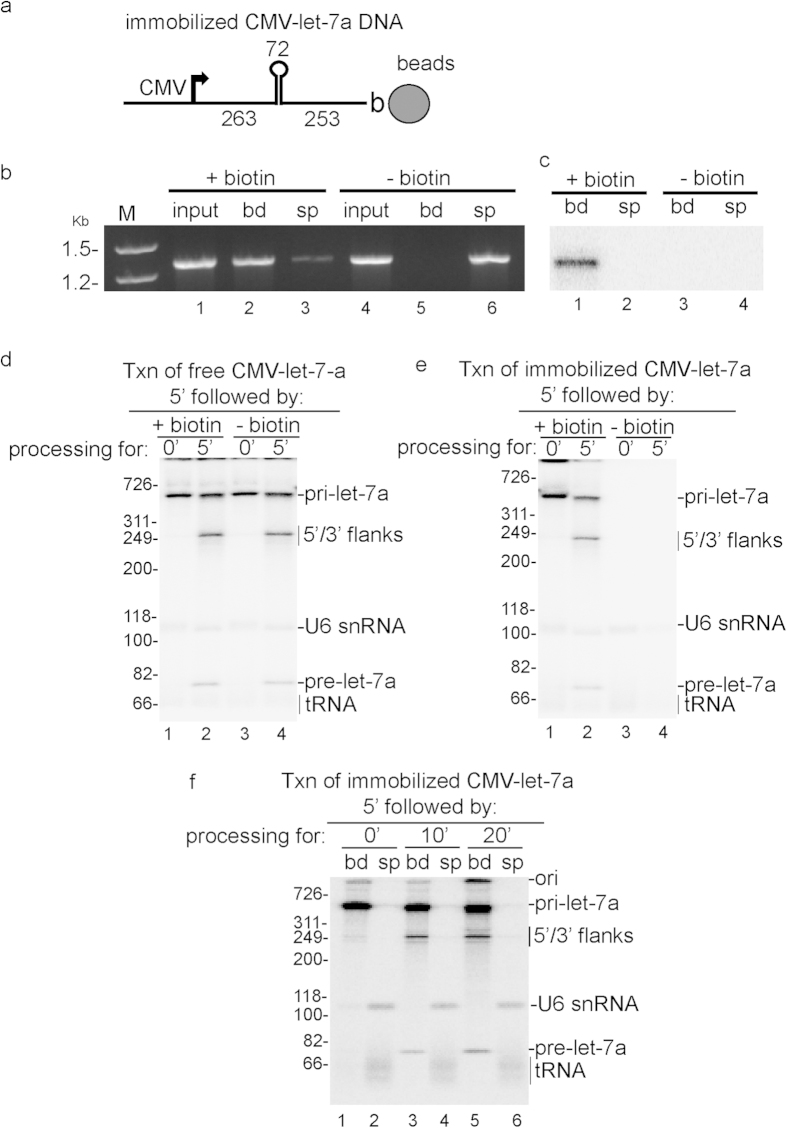
Pri-let-7a miRNA processing occurs on an immobilized DNA template. (**a**) Schematic of CMV pri-let-7a DNA template bound to streptavidin magnetic beads via biotin at the 3′ end. (**b**) CMV pri-let-7a DNA templates containing biotin or no biotin were incubated with streptavidin magnetic beads. DNA templates in bound (bd) and supernatant (sp) fractions were analyzed on a 1% agarose gel by staining with ethidium bromide. (**c**) Biotinylated CMV pri-let-7a DNA template was 3′ end-labeled, bound to streptavidin magnetic beads and incubated in nuclear extract at 30^o^ for 30 min. Bound and supernatant fractions were analyzed on an 8% denaturing polyacrylamide gel. (**d**) CMV pri-let-7a DNA templates containing biotin or no biotin were incubated in nuclear extracts under txn/processing conditions for the indicated times. Total RNA was isolated and fractionated on an 8% denaturing polyacrylamide gel. (**e**) Streptavidin magnetic beads were incubated with CMV pri-let-7a DNA templates containing biotin or no biotin. After washing, beads with or without immobilized DNA templates were incubated in nuclear extracts under txn/processing conditions for the times indicated. Total RNA from each sample was isolated and fractionated on an 8% denaturing polyacrylamide gel. (**f**) Immobilized CMV pri-let-7a DNA template was incubated under txn/processing conditions for the times indicated. The bound and supernatant fractions were separated, and the bound fraction was washed. Total RNA was then isolated from the bound and supernatant fractions and run on an 8% denaturing polyacrylamide gel.

In previous work, we found that pre-mRNA splicing could occur while the CMV-DNA was immobilized on beads^14^. Thus, we investigated whether pri-miRNA processing can occur on an immobilized DNA template (see schematic, Fig. 4a). The CMV DNA template encoding let-7a pri-miRNA was amplified using a 3′ primer that was end-labeled with biotin and a CMV let-7a pri-miRNA DNA template lacking biotin was used as a negative control. As shown in Fig. 4b, only the biotinylated PCR product (lanes 1–3) but not the non-biotinylated PCR product (lanes 4–6) bound efficiently to the streptavidin beads. Moreover, the biotinylated^32^, P-labeled CMV-let-7a DNA template did not detach from the beads when they were incubated for 30 min in nuclear extract under coupled txn/pri-miRNA processing conditions, as indicated by the presence of the labeled, biotinylated DNA template only in the bound, but not the supernatant fraction (Fig. 4c).

To determine whether the biotionylated CMV-DNA construct was functional in our system, we carried out the coupled txn/pri-miRNA processing reaction using the DNAs free in solution. As shown in Fig. 4d, both RNAP II transcription and pri-miRNA processing were not affected by the presence of biotin (compare lanes 1 and 2 to 3 and 4). We then carried out the same reaction using the immobilized let-7a DNA template, which revealed that pri-miRNA processing retains its efficiency on the immobilized template (Fig. 4e, lanes 1 and 2). As expected, when a DNA template without biotin (-biotin) was used as a negative control, only U6 snRNA and tRNA from the nuclear extract were detected but not the CMV transcript (Fig. 4e, lanes 3 and 4).

After optimizing this system via the studies presented in Figs 4 a–e, we used it to ask whether pri-miRNA processing could occur while the nascent transcript was associated with the immobilized template or only after release from the template. To do this, we incubated the beads containing the immobilized CMV DNA construct encoding let-7a pri-miRNA under coupled txn/pri-miRNA processing conditions. The beads and supernatant were then separated followed by extensive washing of the beads and analysis of the total RNA in the bound and supernatant fractions (Fig. 4f). As expected, after 5 min transcription and without further incubation, the full-length transcript was detected, and it was present only in the bound fraction (lane 1). In contrast, the tRNA and U6 snRNA were present in the supernatant (lane 2). Significantly, with longer incubation (10 and 20 min), the 5′ and 3′ flanks and pre-miRNA were detected in the bound fraction but not in the supernatant whereas the U6 and tRNA were only present in the supernatant (lanes 3–6). These data indicate that processing can occur while pri-miRNA is still tethered to the DNA template, consistent with studies reporting co-transcriptional processing of pri-miRNA *in vivo*^25^,^26^,^27^,^28^. The observation that the flanking regions and pre-miRNA are present in the bound fraction suggests that there is an association between the pri-miRNA processing machinery and RNAP II.

In previous work, others and we developed robust *in vitro* systems for coupling RNAP II transcription to pre-mRNA splicing^14^,^36^,^37^ as well as a highly efficient 3-way system for coupling RNAP II transcription to splicing and polyadenylation^15^,^16^,^17^. Biochemical studies using the coupled txn/splicing system led to important insights into the mechanisms involved in coupling^14^,^38^ and the coupled txn/pri-miRNA processing system reported here will likewise be important for determining the mechanisms for this coupling. Our work, in conjunction with the *in vivo* studies on coupling between transcription and pri-miRNA processing, add to the growing list of gene expression steps that are functionally coupled. The dramatic increase in both the rate and efficiency of pri-miRNA processing observed in our coupled system underscores the fundamental importance of this coupling event in gene expression.

## Methods

### Plasmids

A 395-nt region surrounding pre-let-7a-1 was amplified from HeLa cell genomic DNA using let f: 5′-ATAGGGCCCTAACTTCATTTTCACGTAA-3′ and

let_r: 5′-AGCGATATCAGTGTACTTGCTACAGACT-3’. Δpre-let-7a was generated by PCR to delete the 72 nt hairpin using the following primers Δpre_f:

5′-TCTCTTCACTGTGGGCTAACGTGATAGAAAAGTCTGCATCCAGGCGGTC-3′ and Δpre_r:

5′-GACCGCCTGGATGCAGACTTTTCTATCACGTTAGCCCACAGTGAAGAGA-3′.

The PCR products were inserted into pcDNA3.1 (-) using ApaI and EcoRV to generate CMV DNA templates encoding let-7a pri-miRNA or Δpre-let-7a pri-mRNA. Subsequently, CMV_f: 5′-TGGAGGTCGCTGAGTAGTGC-3′ and CMV_r:

5′-TAGAAGGCACAGTCGAGG-3′ were used to amplify PCR products containing both CMV and T7 promoters from the vectors. The resulting PCR products were purified and used for transcription with RNAP II or T7 RNA polymerase. Let-7a pri-miRNA transcribed from the CMV promoter contains 263 nt upstream of the 5′ cleavage site and 253 nt downstream of the 3′ cleavage site of the 72 nt let-7a pre-miRNA hairpin. Let-7a pri-miRNA generated by the T7 promoter from the same PCR product is identical except for 53 nt of vector sequence that is not present at the 5′ end. pcDNA3-miR-21 (Plasmid #21114) and pcDNA3-miR26 (Plasmid #21115) were described^39^ and obtained from addgene.

### Coupled RNAP II txn/pri-miRNA processing system ***in vitro***

HeLa nuclear extract was prepared as described^34^ except that the final centrifugation following dialysis was omitted, which results in increased efficiency of RNAP II transcription^14^. For assembly of PICs, the pri-miRNA DNA templates (4 μls containing 800 ng DNA) were incubated with 1 μl MgCl_2_ (80 mM), 3 μl 15% polyvinyl alcohol (PVA) and 15 μl HeLa nuclear extract at 30 °C for 20 min. To initiate RNAP II txn, 1 μl ^32^P-UTP (800 Ci/mmol; Perkin Elmer Life Sciences), 1 μl ATP (12.5 mM) and 1 μl creatine phosphate (500 mM, di-Tris salt) were added to the reaction mixture. After transcription for 5 min at 30 °C, 5 μl of the reaction mixtures were placed in a new tube and incubated with 18.2 μl nuclear extract and 1.8 μl 80 mM MgCl_2_ (final 6.4 mM) for pri-miRNA processing at 37 °C for times indicated in each figure. Processing of T7 let-7a pri-miRNA was performed under txn/processing conditions except that DNA templates and ^32^P-UTP were omitted and RNAs were directly added to the reaction mixtures after incubation under PIC formation conditions. For processing of naked CMV pri-let-7a (Fig. 3d), RNAP II txn was carried out for 5 min, and total RNA was extracted from the reaction with Trizol (Invitrogen) and then added to a txn/processing reaction as described above for the naked T7 RNA. To control for the possibility that the total RNA present in the nuclear extracts may inhibit processing of naked pri-let-7a transcript, total RNA was extracted from a mock txn reaction without DNA template, and this RNA was added together with CMV-let-7a DNA and ^32^P-UTP to carry out the txn/processing reaction. Total RNA was then isolated using Trizol and analyzed on 8% polyacrylamide, 8 M urea denaturing gels.

### Oligonucleotide-directed RNase H cleavage assay

For RNase H cleavage of naked RNAs, total RNA was isolated from an aliquot (5 μl) of a txn/processing reaction after processing for 10 min. The naked RNA was resuspended in a 25 μl RNase H reaction mixture containing 20 pmol of a DNA oligonucleotide (designated X, Y or Z), 12 mM Hepes, pH 8.0, 60 mM KCl, 3 mM MgCl_2_, 1 mM DTT, 0.5 μl RNasin (40 U/μl, Promega), and 1 U of E. coli RNase H (Invitrogen). For RNase H cleavage of RNA in the txn/processing reaction, 20 pmol of the DNA oligonucleotide was directly added to 5 μl after completion of the txn/processing reaction followed by continued incubation for 10 min. Total RNA was then isolated and detected. The sequences of the oligos used for RNase H cleavage are as follows: X: 5′-ACTTACGTGAAAATGAAGTT-3′; Y: 5′-TTATTTCCAGGCCATAAACA-3′; Z: 5′-CTCCCAGTGGTGGGTGTGACCC-3′.

### RNAP II txn/pri-miRNA processing on immobilized DNA template

Biotin labeled PCR products were generated using CMV_f and biotin labeled CMV_r. The biotin was linked to the 5′-end oligonucleotide via a 15-atom spacer (TEG, Operon) as described^18^. 200 ng biotinylated CMV-DNA templates were bound to 10 μl of streptavidin magnetic particles (Roche) for 30 min and washed according to the manufacturer’s protocol. Immobilized CMV-DNA templates bound to the beads were then used for RNAP II transcription and pri-miRNA processing as performed with free CMV DNA templates. Bound and supernatant fractions were separated using a magnetic separator. Bound fractions were washed with 1 ml wash buffer (10 mM HEPES pH 7.6, 10% glycerol, 0.1 mM EDTA and 50 mM KCl) three times before RNA extraction.

## Additional Information

**How to cite this article**: Yin, S. *et al.* Primary microRNA processing is functionally coupled to RNAP II transcription *in vitro*. *Sci. Rep.* **5**, 11992; doi: 10.1038/srep11992 (2015).

## Supplementary Material

Supplementary Information
