# Supplementary material for: Primary microRNA processing is functionally coupled to RNAP II transcription in vitro
Source: Sci Rep. 2015 Jul 7;5:11992. doi: 10.1038/srep11992 (PMC4493704; doi:10.1038/srep11992)
Supplement: Supplementary Information [file srep11992-s1.pdf]

Supplementary Information

Title:

Primary microRNA processing is functionally coupled to RNAP II transcription in vitro

Authors:

Shanye Yin, Yong Yu and Robin Reed

Supplementary Information

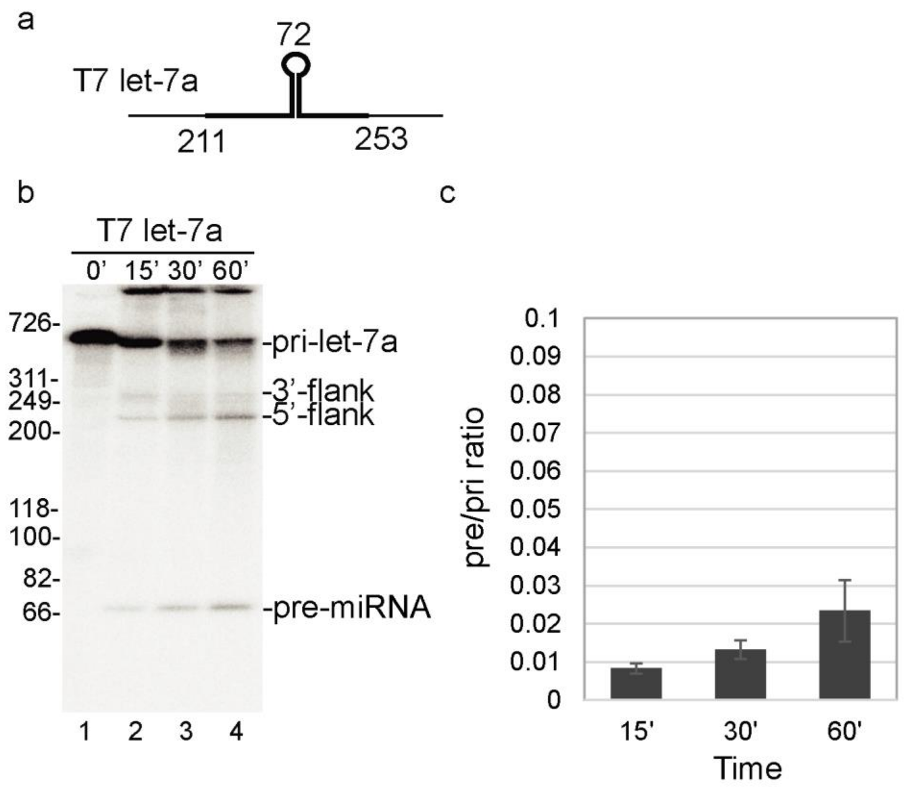

Supplementary Figure S1. Pri-miRNA processing of naked T7 let-7a pri-miRNA in vitro. (**a**) Schematic showing the T7 let-7a pri-miRNA transcript used for in vitro processing. (**b**) T7 let-7a pri-miRNA was incubated with nuclear extract in the presence of 6.4 mM $MgCl_2$ for the indicated times. Total RNA was isolated, run on an 8% denaturing polyacrylamide gel, and bands detected by phoshorimager. (**c**) The percentage of T7 let-7a pri-miRNA processed at each time point in panel C was determined as the amount of pre-let-7a relative to pri-mRNA at the start of the reaction. RNAs were detected by phosphorimager and quantified using Quantity One software. Mean ± S.D. of three biological replicates are shown.

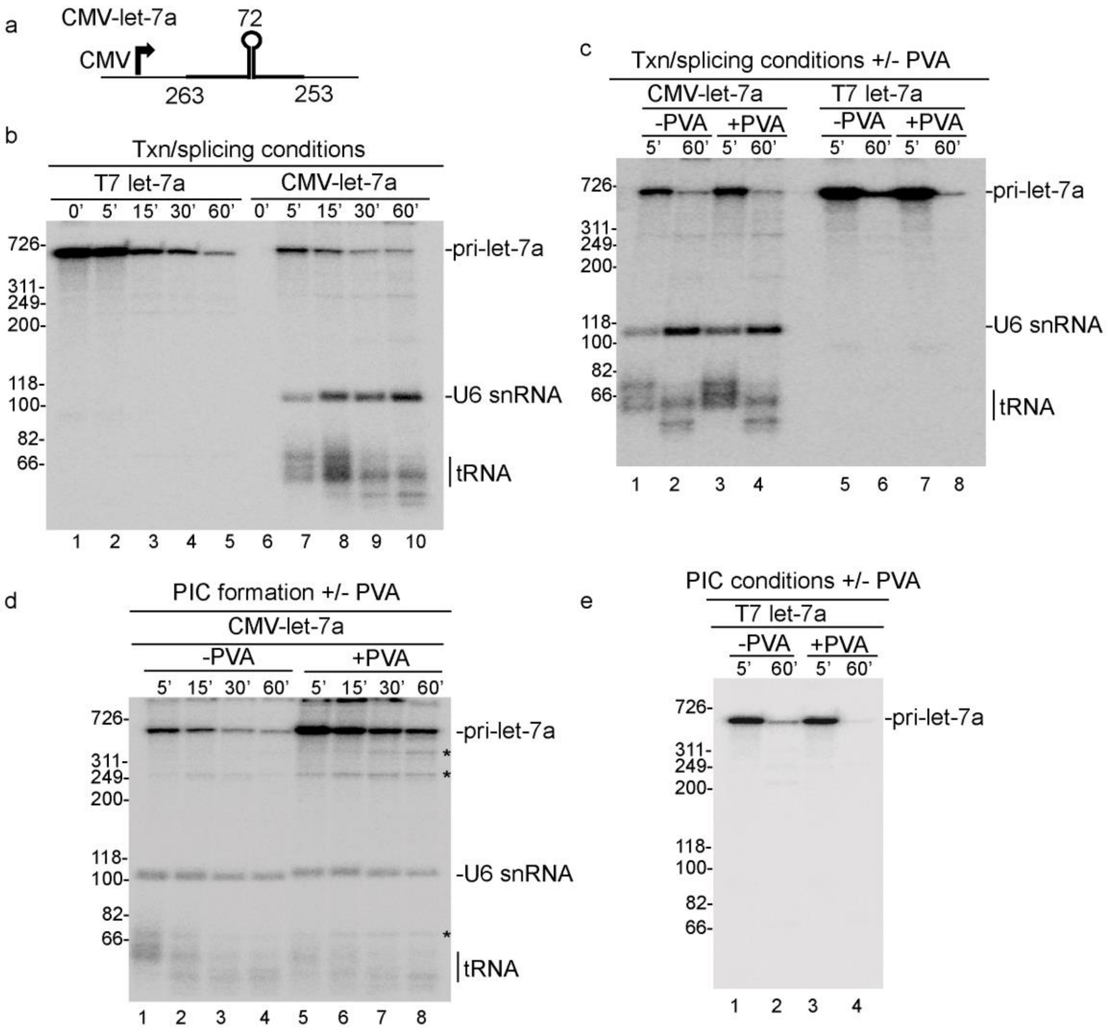

Supplementary Figure S2. Optimization of RNAP II txn/pri-miRNA processing conditions. (**a**) Structure of the DNA template encoding CMV-let-7a pri-miRNA used to optimize RNAP II transcription and pri-miRNA processing. The sizes of the 5' flank, pre-miRNA, and 3' flank are shown. (**b**) T7 let-7a pri-miRNA or CMV DNA template encoding let-7a pri-miRNA was incubated with nuclear extract under txn/splicing conditions (with 3.2 mM $MgCl_2$), for the indicated times. Total RNA was isolated and run on an 8% denaturing

polyacrylamide gel. The asterisks indicate bands that could correspond to the flanking regions (top asterisks) and pre-miRNA (bottom asterisk) based on their sizes. (**c**) T7 let-7a pri-miRNA or CMV DNA template encoding let-7a pri-miRNA was incubated with nuclear extract under txn/splicing conditions (with 3.2 mM $MgCl_2$), with or without PVA for the indicated times. In the coupled reaction, α-amanitin was added (**d**) CMV DNA template encoding let-7a pri-miRNA was incubated under PIC conditions in the presence or absence of PVA followed by continued incubation for the times indicated under txn/splicing conditions (with 3.2 mM $MgCl_2$). (**e**) Same as D except using T7 let-7a pri-miRNA. For the coupled reactions shown in panels b-d, -amanitin was added at the 5-minute time point to block further transcription, followed by continued incubation to allow processing. The endogenous U6 snRNA and tRNA that are present in the nuclear extract and labeled in the reaction are indicated.

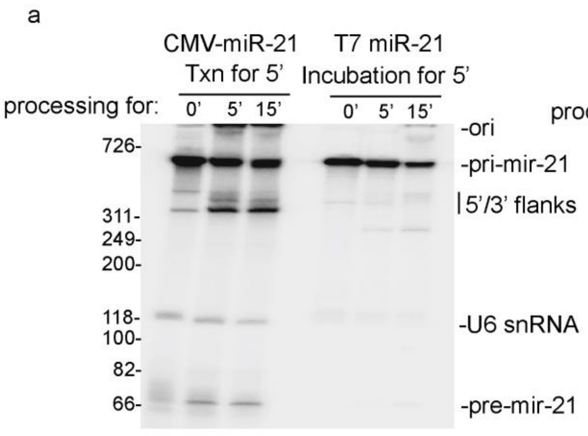

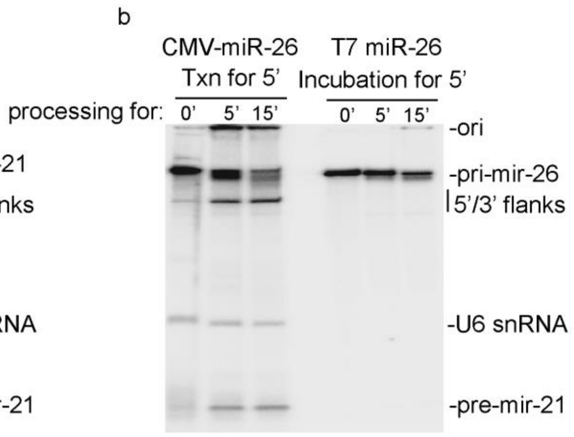

Supplementary Figure S3. Coupled txn/pri-miRNA processing using two different miRNAs. (**a, b**) CMV DNA templates encoding miR-21 pri-miRNA (**a**) or encoding miR-26 (**b**) pri-miRNA or the corresponding T7 transcripts were incubated in nuclear extracts in the txn/pri-miRNA processing system for the indicated times. Total RNA was isolated and fractionated on an 8% denaturing polyacrylamide gel. The RNA species are labeled and ori indicates the gel origin.
